# Fluid-solid phase transition of n-alkane mixtures: Coarse-grained molecular dynamics simulations and diffusion-ordered spectroscopy nuclear magnetic resonance

**DOI:** 10.1038/s41598-018-37799-7

**Published:** 2019-01-30

**Authors:** S. Shahruddin, G. Jiménez-Serratos, G. J. P. Britovsek, O. K. Matar, E. A. Müller

**Affiliations:** 1 PETRONAS Research Sdn. Bhd, Lot 3288 & 3289 Off Jalan Ayer Itam, Kawasan Institusi Bangi, 43000 Kajang, Malaysia; 2 Department of Chemical Engineering, Imperial College London, South Kensington Campus, London, SW7 2AZ UK; 3 Department of Chemistry, Imperial College London, South Kensington Campus, London, SW7 2AZ UK

## Abstract

Wax appearance temperature (WAT), defined as the temperature at which the first solid paraffin crystal appears in a crude oil, is one of the key flow assurance indicators in the oil industry. Although there are several commonly-used experimental techniques to determine WAT, none provides unambiguous molecular-level information to characterize the phase transition between the homogeneous fluid and the underlying solid phase. Molecular Dynamics (MD) simulations employing the statistical associating fluid theory (SAFT) force field are used to interrogate the incipient solidification states of models for long-chain alkanes cooled from a melt to an arrested state. We monitor the phase change of pure long chain n-alkanes: tetracosane (C_24_H_50_) and triacontane (C_30_H_62_), and an 8-component surrogate n-alkane mixture (C_12_-C_33_) built upon the compositional information of a waxy crude. Comparison to Diffusion Ordered Spectroscopy Nuclear Magnetic Resonance (DOSY NMR) results allows the assessment of the limitations of the coarse-grained models proposed. We show that upon approach to freezing, the heavier components restrict their motion first while the lighter ones retain their mobility and help fluidize the mixture. We further demonstrate that upon sub-cooling of long n-alkane fluids and mixtures, a discontinuity arises in the slope of the self-diffusion coefficient with decreasing temperature, which can be employed as a marker for the appearance of an arrested state commensurate with conventional WAT measurements.

## Introduction

There is extensive literature describing wax deposition as one of the most important flow assurance issues in the petroleum industry^1–4^. Waxy crude oil contains a disproportionate amount of high molecular weight linear alkanes, which are generally soluble in the crude oil at reservoir conditions. However, upon production and transport (particularly at sub-sea conditions), the temperature will drop and the solubility of these alkanes will decrease drastically, inducing precipitation and, subsequently, formation of obtrusive, hard deposits^5^. While this is by no means a new problem^6–8^, the detrimental effects of wax deposition encountered during production, transportation, processing and storage cause significant financial losses through the cost of remedial operations^9,10^.

The accepted deposition mechanism recognizes that n-alkanes with 18 or more carbon atoms will form crystalline solid substances at around 20 °C or below, known generically as ‘waxes’. The amount and chain length distribution of parent n-alkanes contained in crude oil varies depending on the source and origin of the crude oil but generally corresponds to a wide multi-component distribution. When the temperature of the solution is lowered sufficiently, molecular motion becomes increasingly hindered causing the molecules to self-assemble forming aligned clusters of chains. This nucleation process continues until the clusters reach a critical size and become stable. Further aging of the nuclei may involve arrangement into crystalline (ordered) structures^11^. Additional molecules may then position themselves on the nucleation sites, or surfaces, and become part of the growing structure which, if left untreated, might eventually block the flow. While the general physical picture described here is fairly accepted, detailed understanding of the molecular mechanism remains elusive. In spite of their apparent chemical simplicity, the behaviour and interactions of n-alkanes with other components of the crude oil, the effect of polydispersity of the mixture, and the effect of added solvents and/or inhibitors are amongst many questions surrounding this phenomenon.

A key quantity defining the above process is the wax appearance temperature (WAT), loosely defined as the temperature where alkane crystals are macroscopically observed upon decreasing the temperature, and is implicitly related to the point of incipient formation of a solid phase. Although the WAT is not a well-defined property from a thermodynamic point of view, as some degree of sub-cooling is commonly required to macroscopically detect a phase change in a finite time, it remains, nevertheless, a key element for thermodynamic models^12^. WAT measurements, in general, are based on the monitoring of the change in a physical property of the oil during the formation of alkane crystals. In finding the WAT, wax formation is first induced by a controlled cooling of the oil, and, as the temperature approaches a state where alkane crystals are being formed, the physical changes that occur with it can be captured by suitable techniques^3^. WAT is commonly determined by standard protocols such as ASTM D250^13^, relying on visual observation of oil cloudiness at the bottom of a test jar. These methods are time-consuming and require operator interactions, which are highly subjective. A further problem arises when considering crude oils where the opacity of the sample renders optical methods difficult to use. A variety of other direct and indirect characterisation techniques are routinely employed including Differential Scanning Calorimetry (DSC)^12,14–16^, viscosimetry^17,18^, scanning under cross polarized microscope^1,16,19,20^, Fourier Transform Infrared Spectroscopy^20–23^, near-infrared scattering^24^, amongst others. A related property, the pour point temperature (ASTM D-97) is also frequently monitored as related to the incipient formation of gels. These techniques all have very specific conditions in which they excel^3,23,25^ but in general, many factors conspire against the universal application of any of the above mentioned techniques: few of them are capable of detecting the actual temperature point that characterizes the phase transition between a homogeneous fluid and the underlying solid phase^23^.

The liquid-solid transition is a first-order physical phase transition, and as such, one would expect that the monitoring of macroscopical thermophysical properties, such as density, viscosity, etc. should be enough to unequivocally pin-point it. The reality is that n-alkane crystallization is a very slow process, sometimes taking weeks to occur. The dynamics may also be masked by the degree of sub-cooling of the sample, and a balance between thermodynamic equilibrium and kinetic driving forces determine the eventual phase transition^26^. Useful insights are gained if one turns to molecular-level interrogations to reduce the characteristic times and enhance the reliability of the results. In this sense, the monitoring of diffusion coefficients^27^ provides direct access to the mechanism involving the arrestment of the fluid and the incipient formation of a solid phase. The development of two-dimensional Diffusion Ordered Spectroscopy (DOSY) Nuclear Magnetic Resonance (NMR) based on pulsed field gradient sequences is a powerful tool to analyse complex and polydisperse samples. NMR studies of petroleum samples have been used to gain insight into physicochemical properties^28^ and/or chemical composition^29^. da Silva Oliveira *et al*. used DOSY NMR to study the behaviour of asphaltenes in solution, in which information on the type of interaction between clusters corresponding to the composition was obtained successfully^30^. This method is also used by Durand *et al*.^31^ with the aim of developing a correlation between structural properties and aggregation behaviour of asphaltenes. More recently, DOSY NMR combined with multiway statistical analysis was reported to demonstrate effectively that the structures and sizes of asphaltenes are closely related, irrespective of their origin^32^. To the best of our knowledge, we are unaware of DOSY NMR being used to study WAT.

The study of the mechanism of crystallization requires a fundamental exploration of the process at the molecular level, which is difficult to obtain from experiments. In this scenario molecular dynamics simulation can be a useful tool, providing insights on the primary nucleation and the crystal growth process. While the behaviour of long chained alkanes has been described extensively through simulations^33–36^, the fundamental process behind the freezing transition is much less reported. Studies are challenged by the fact that while crystallization is a process that spans several length and time scales, classical simulation techniques have a spatial and temporal resolution of only a few nanometers and nanoseconds. Although recent advances in molecular modelling, mainly employing coarse-graining strategies, have reduced the limitations associated with atomistic models^37,38^, they, nevertheless, remain insufficient to study crystallization fully in complex multicomponent mixtures.

In this contribution, we probe the incipient formation of the emerging solid phase by monitoring the diffusion coefficients, using coarse-grained (CG) molecular dynamics (MD). Separately, we show that the measurement of diffusion coefficients through DOSY NMR is an unambiguous method for measuring the WAT (or, in effect, the liquid-solid phase change boundary), superior to conventional experimental approaches. Finally, we use a combination of MD and DOSY NMR approaches to study the appearance of a solid phase upon cooling in a synthetic model mixture, whose properties mimic closely those of field samples of typical waxy crudes. Our study provides insight into the liquid-solid transition in heavy molecular weight n-alkanes, which can be exploited to design wax-inhibiting chemicals for effective remedial strategies in the oil industry.

## Results

### Comparison of MD and NMR data

**Figure 1:**
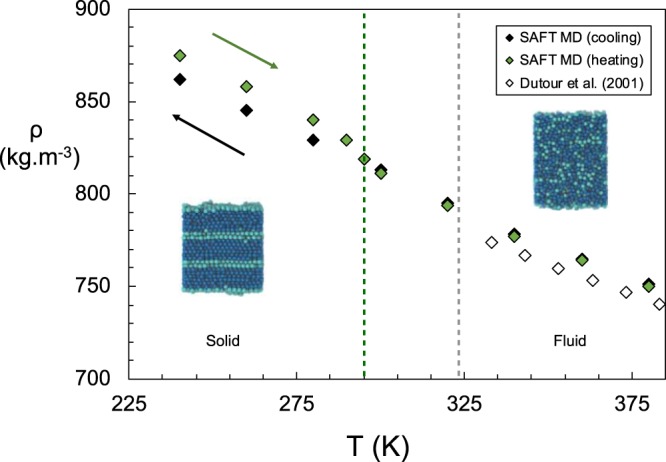
Density of pure n-C_24_H_50_ as a function of temperature. Green and black diamonds correspond to results from simulations of melting of a pre-assembled crystal, and cooling from a melt, respectively; the open diamonds represent experimental data^63^. The dashed grey line indicates the experimental melting point (WAT) measured by differential scanning calorimetry; the dashed green line indicates the melting point of the coarse-grained SAFT model. Inserts show snapshots of a pre-formed structured solid phase used to initiate the heating runs (left) and a snapshot of a high-temperature fluid state (right).

Pure alkanes are employed to evaluate the performance of the molecular models. Results are presented here for C_24_H_50_, as it is an archetypical long n-alkane; other similar results (*e*.*g*. C_30_H_62_) are provided in the Supplementary Information (SI) section. Figure 1 presents the density behavior in the vicinity of the solid-fluid transition as obtained from direct MD simulations using a CG force field^39^ based on the statistical associating fluid theory (SAFT). Molecular models fitted to the properties of fluids (as opposed to solids) struggle to provide accurate predictions of the melting points as they fail to reproduce the correct crystal structures. Even refined atomistic models of n-alkanes show deviations of 10 to 40 K from experimental data^40^. However, the solid-fluid transition of a given molecular model may be explored, by heating a pre-formed crystal until its melting is detected^41^. Figure 1 shows that for the SAFT model, this transition corresponds to 295 ± 5 K. Decreasing the temperature, starting from a liquid state, simulation models will typically fail to detect the formation of a crystal phase and/or the melting point, but will explore the metastable liquid states instead^42^. The large energy barriers for nucleation, seen even with the smaller alkanes^43^, provide computational challenges to study the formation of crystal structures, and preclude the formation of a stable crystalline solid within the limits of the available simulation times. It is seen that the configurational properties of the chains, such as the density, the end-to-end distances and radii of gyration calculated from the MD do not change their temperature dependence even past the expected phase transition (see SI); that is, there is no apparent change in the behavior of the fluid even upon significant supercooling below the melting point. Similar results are obtained for C_30_H_62_ and other pure alkanes studied.

**Figure 2:**
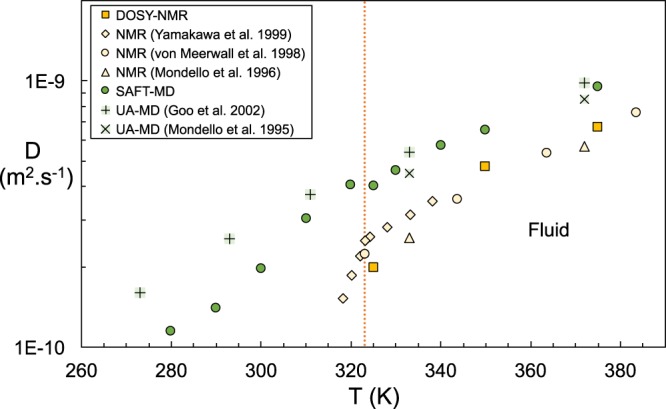
Diffusion coefficients of pure n-C_24_H_50_ as a function of temperature obtained from DOSY NMR and coarse-grained MD simulations using the SAFT force field. Comparison is made with MD data using united atom (UA) models^45,47^, and PFGSE NMR^44,46,48^. The dashed red line indicates the melting point measured by DSC.

Figure 2 compares the self-diffusion values for C_24_H_50_ obtained via MD simulation using CG SAFT force fields, and determined experimentally through DOSY NMR; also included in Fig. 2 are reported literature^44–48^ values found along a cooling trajectory over a range of temperatures from 420–250 K. A similar plot for C_30_H_62_ is provided in the SI. The diffusion coefficients determined experimentally by NMR methods obtained in this work are consistent with those reported by Yamakawa *et al*.^46^, Mondello *et al*.^44^ and von Meerwall *et al*.^48^ for C_24_H_50,_ (and those of Vardag *et al*.^49^ for C_30_H_62_). The higher frequency NMR used in this work allows for a higher resolution and the acquisition of well-resolved spectra which contribute to an increased confidence in the diffusion values. DOSY employs an improved pulse sequencing which helps to further deconvolute the signals that are overlapped, which again can significantly improve the spectral resolution^50,51^.

For C_24_H_50_ the fluid-solid transition is determined from differential scanning calorimetry (DSC) to be 323 K (see SI for the DSC results) consistent with the reported literature values in the range of 323–327 K^52^. At this point, the data of Yamakawa *et al*.^46^ presents a change in the slope of the temperature dependence, presumably a marker of the presence of the rotator phase, which ends at 320 K at the rotator-crystal transition temperature. The DOSY-NMR signal vanishes at lower temperatures, indicating an arrestment of detectable molecular motion and the existence of a solid state. Similarly, we were not able to detect NMR signals for C_30_H_62_ below 340 K corresponding to its melting point, reported between 335–338 K^52^.

**Figure 3:**
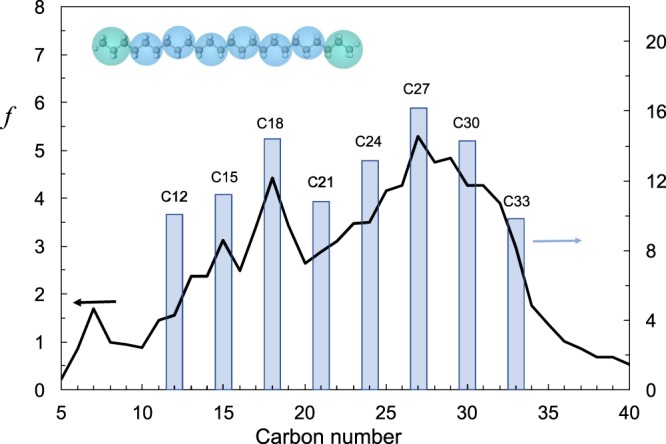
n-alkane distribution *f* (mass %) of a typical Malaysian reservoir oil obtained by high pressure gas chromatography (solid line, left axis). Bars correspond to the mass % distribution of a surrogate mixture composed of pure n-alkanes (right axis). The mass weighted average of both distributions corresponds to n-C_24_H_50_. Cartoon depicts the SAFT coarse-grained model composed of eight beads for C24.

Freezing by a nucleation event, which is an activated process, is not seen in our simulations. The stochastic nature of nucleation makes the determination of nucleation rates from a finite-size simulation an unreliable process due to the sheer unlikelihood of the event. Little is known about the free energy and/or structure of the critical nuclei, both of which are key properties to understand the freezing of chain molecules. Molecular simulation studies can only shed light on the incipient stages of the formation of the crystalline phase and allow us to study only the first stages of the progression towards solidification facilitated by the decrease in temperature. We compare our results of cooling from a liquid state with simulation models for n-alkanes reported in the literature corresponding to higher-fidelity united-atom (UA) models, where a unique force center is employed for each carbon center and its associated hydrogen atoms^38,41,53,54^. At this level of description, diffusion coefficients have been favorably compared to NMR results for smaller alkanes^55^. The apparent simplicity of the SAFT force fields used herein, which groups three carbons and their associated hydrogens in each molecular bead (*c*.*f*. insert of Fig. 3) does not hinder their reliability. Despite the coarse-grained description of the potentials in the SAFT models, their prediction of the diffusion values for both C_24_H_50_ and C_30_H_62_ are in good agreement with those previous simulations while providing a speedup of over an order of magnitude in computational performance. Neither atomistic nor CG models are in quantitative agreement with the experimental results, showcasing a limitation in terms of their direct employment as replacements for experimental evaluation of the melting point. Furthermore, they both overestimate the diffusivity. This discrepancy is common to coarse grained simulation models and is attributed to the smoothing out of the intermolecular interactions by the sheer nature of the simplified free energy landscape.

A change in the slope of the diffusion coefficient with temperature is observed for the CG simulations at temperatures below the expected freezing point of the model. This change in slope suggests a slow-down of the fluid dynamics and appears at state points where a solid would be expected to be the stable coexisting phase. While we make no claim as to the quantitative accuracy of this behavior, it is a reproduceable trait and suggests that in small size systems and short times (as compared to macroscopic scales and times), the molecules experience an arrestment in their mobility, consistent with current theories of glass-forming fluids.

### 8-component alkane mixture

An alkane mixture consisting of 8 n-alkanes, ranging from C_12_H_26_ to C_33_H_68_ (details on the mixture composition are given in the SI), inspired by the composition of a Malaysian oil, is employed as a surrogate of a waxy crude (see Fig. 3). Other components in the crude (e.g. resins, asphaltene molecules), which inevitably have an influence on the WAT^56^, are excluded from this study.

**Figure 4:**
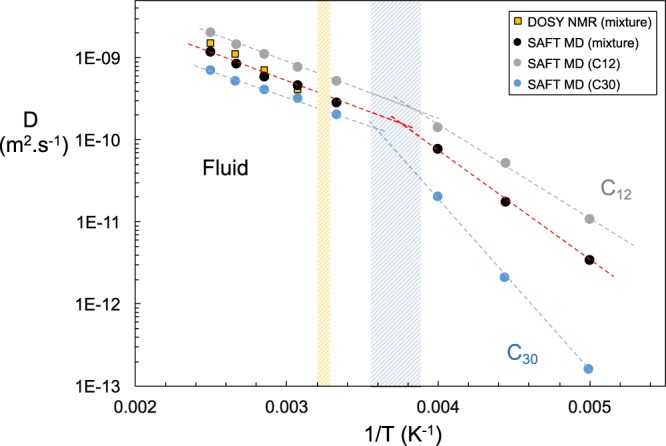
Arrhenius plots of the self-diffusion coefficients (D) of the 8-component alkane mixture determined via coarse-grained MD simulation (black circles) and DOSY NMR (yellow squares). Grey and cyan symbols are the diffusion coefficients of C_12_H_26_ and C_30_H_62_ in the mixture, respectively. Dashed lines are linear fits to the data in the fluid and arrested regions. Shaded blue region is the expected melting transition as suggested by the simulation model; shaded yellow delineates the melting region of the experimental mixture as determined from DSC.

The system diffusivity as a function of the inverse of the temperature is shown in Fig. 4. It is observed that the average diffusion coefficients predicted from MD simulations follow the qualitative trend obtained from DOSY NMR although the Arrhenius plot suggests a slightly different activation energy. A change in the slope of the diffusion data upon cooling, which corresponds to the retardation in the rate of molecular motion, is indicative of a phase change. This sudden decrease in diffusion coefficient as the system crosses the phase transition has been reported in the literature^57^. Results for the diffusion coefficients of the individual components within the mixture are also calculated, and we present the results of the smallest alkane and a representative long alkane. No NMR signal is detected below 300 K suggesting this to be an upper limit to the WAT. The definition of the melting point of the mixture is ambiguous, as the large size differences between the molecules support the staggered freezing of the individual components. DSC results for the mixture (see SI) suggest a similar ambiguity in terms of a wide band of about 20 K where the melting process occurs.

## Discussion

Despite the fact that the sub-cooling of the model fluids below their melting point does not produce any sharp morphological change, the mean square displacement of the atomic centers of the molecules, related to the self-diffusion coefficients (or the related Lindemann index^58^) can be employed as a sensitive marker for the phase transition. An Arrhenius plot of the self-diffusion coefficient of a subcooled liquid shows a change in slope that coincides with the expected phase change. This cessation in the motion of fluid is not to be confused with the glass transition (which for n-alkanes is in the range 105–138 K^59^ and seems to suggest the onset of a behavioral change of the subcooled liquid where parts of the system become increasingly spatially-correlated^60^. The exact nature of these heterogeneities and the question of how, or if they relate to the structure, has been a matter of controversy^61^ and is beyond the scope of this communication. We have used this decrease in the mean square displacement as a marker for identifying the potential for the appearance of a solid phase, as it coincides with the solid-fluid transition of the model, without making any claims with respect to the quantitative correlation of the results. The close monitoring of diffusion allows the study of the initial events that trigger the wax appearance upon cooling without the need of making any assumption on the morphology of the emerging solid crystal phase, and is a unique aspect of the methodology proposed.

Although the average diffusivity of the mixture (obtained either through DOSY NMR or simulations) suggests a unique transition point, simulations allow one to further examine the dynamic behavior of the individual components present in the mixture. Analysis of the individual diffusion coefficients of the various n-alkanes in the mixture shows that the smallest or lightest component, C_12_H_26_, moves significantly faster than other analyzed compounds even at low temperatures. The heavier alkanes, C_24_H_50_ and C_30_H_62_, were observed to have lower diffusion rates, reflecting their larger sizes, as well as higher molecular weights. This is an expected result for the fluid phase of alkane mixtures, which can be rationalized by scaling arguments^62^, where the diffusion of alkane mixtures is seen to experimentally be proportional to 1/*N*^2^, where *N* is the chain length^43,63^. The scaling is also applicable to mixtures in the fluid region, hence the value of the overall diffusion coefficient is a mass-weighted average of the individual components as pointed out by von Meerwal *et al*.^43^ and Alatas *et al*.^64^. An important observation is that the WAT (or freezing) is not a unique temperature point, but rather a range of well-defined temperatures, initiated by the slowing down of the heavy components, and maintained by the fluidity of the lighter components, *i*.*e*. the analysis of the individual alkanes hints that it is the heavier alkanes that initiate and dominate the phase transition while lighter alkanes remain mobile at lower temperatures and are the last of the components to arrest their movement. The results cannot be directly deduced from the pure component properties; the heavier components C_24_H_50_ and C_30_H_62_ have slightly lower diffusivity in the mixture than in the neat form.

The results associated with the 8-alkane mixture model system suggest the formation of an arrested (gel) state upon a decrease in the temperature. The coexistence in a mixture of very asymmetric compounds is a recipe for these gels to form rapidly and there is evidence that this gelation process is the precursor stage of the crystallisation process. Morozov *et al*.^65^ observed the formation of gel when the temperature of crude oils is reduced below the WAT, and showed that the deposition of wax can be accelerated with significant temperature decrease. Similar findings were reported by Dimitriou *et al*.^66^ describing a change in the yield stress and viscosity during the transition towards gel formation. It is based, on this evidence, that we suggest that the arrested states have some gel-like character. In this context, the term gel is used rather indistinctively from the term “metastable sub-cooled liquid”, as there is no measurable quantity (other than diffusion) to differentiate between them.

From a field operations point of view, the molecular picture suggests that the coupling of wax inhibitors to the more mobile (e.g. aromatics) elements of the crude mixture would serve as an avenue to arrest the incipient formation of solids. Although this concept has been applied empirically, it contrasts with the perceived assumption that wax inhibitors should be composed of heavily branched alkanes which disrupt the formation of ordered wax crystals^67^. Furthermore, as NMR is rapidly becoming a miniaturized and common tool in industry^68^, it could be employed as a non-invasive probe for the *in*-*situ* determination of either WAT or the onset of freezing.

## Methods

### Molecular model

The molecular model used in this study is based on a coarse-grained top-down approach where several closely bonded atoms are represented by a single interaction bead whose parameters are back-traced to the thermophysical properties of small constituent molecules (e.g. small alkanes). A discussion of the SAFT-γ force field is given in a recent review^39^ and in subsequent papers^69–73^. The force field has been employed to model the phase behaviour of complex fluid mixtures^74^, polymers and surfactants^75,76^ and can be applied to qualitatively understand solid-fluid phase behaviour^77^. Within the SAFT-γ formulation, beads interact through a Mie potential (generalised Lennard-Jones potential). The SAFT potential parameters (segment diameter σ, potential energy depth ε, repulsive exponent λ) are commonly fitted to PvT properties of small molecules or are obtained from corresponding states correlations^78^. While there are several published SAFT models for alkanes^70,74^ these have been fit to individual alkanes, are homonuclear in nature and no parameters are provided for the longer alkanes. Rahman *et al*.^73^ suggested a heteronuclear model where three consecutive carbon atoms in an alkane backbone and their associated hydrogen atoms (CH_2_CH_2_CH_2_) are represented by one coarse-grained bead. A differentiation on the terminal groups (CH_2_CH_2_CH_3_) is made to account for better representation, transferability and robustness of the molecular models when studying different types of alkanes. The intramolecular potential parameters were determined from simulations of atomistic united atom of alkane systems with chain lengths 3*n* (for *n* = 1, 2, …). It was assumed that the intramolecular interactions were attributed to bond stretching and bond angle bending. Force field details are provided in the SI.

### Diffusion Coefficient by MD Simulation

Classical Molecular Dynamics (MD) simulations are performed using HOOMD-blue (Highly Optimized Object-oriented Many-particle Dynamics)^79^. Randomly-generated configurations of 1639 molecules (pure alkanes) or 7939 molecules (mixture) are initially equilibrated for at least 10 ns under the isobaric-isothermal ensemble conditions at a pressure of 1 bar and at the desired temperature to generate a stable liquid phase. Following equilibration, a further simulation is performed in the canonical (isochoric-isothermal) ensemble for calculating the diffusion coefficients. The simulations are performed for a minimum 50 ns; longer simulation times were employed at lower temperatures. Specific details are provided in the SI.

Self-diffusion coefficients (*D*) are obtained from the Einstein equation^80^ which relates the time evolution of the mean square displacement (MSD) of each bead of the molecule to the diffusion coefficient. The diffusion coefficient is calculated from the slope in the long-time regime of the MSD curve as a function of time, using the individual CG segment positions along the simulation trajectory. In the SI we present a detailed description of the calculation and details.

### Diffusion coefficients by ^1^H DOSY NMR

DOSY experiments were carried out on a Bruker 500 MHz AVANCE III HD spectrometer running TopSpin3.2 and equipped with a z-gradient bbfo/5 mm tuneable SmartProbe and a GRASP II gradient spectroscopy accessory providing a maximum gradient output of 53.5 G/cm (5.35 G/cmA)^81^. The ^1^H DOSY spectra were collected using the Bruker pulse program ledbpgp2s at a frequency of 500.13 MHz with a spectral width of 5500 Hz (centred on 4.5 ppm) and 32768 data points. A relaxation delay of 12 s was employed along with a diffusion time (large delta) of 150 ms and a longitudinal eddy current delay (LED) of 5 ms. Bipolar gradients pulses (little delta/2) of 5 ms and homospoil gradient pulses of 1.1 ms were used. The gradient strengths of the 2 homospoil pulses were −17.13% and −13.17%. 24 experiments were collected with the bipolar gradient strength, initially at 2% (1^st^ experiment), linearly increased to 95% (24^th^ experiment). All gradient pulses were smoothed-square shaped (SMSQ10.100) and after each application a recovery delay of 200 µs used. The data was processed using 16384 data points in the direct dimension applying an exponential function with a line broadening of 1 Hz and 128 data points in the indirect dimension. Further processing was achieved using the Bruker Dynamics Center software (version 2.1.7) – error estimation by Monte Carlo simulation with a confidence level of 95%^82^.

## Supplementary information

Supplementary Material
