# Supplementary material for: Fluid-solid phase transition of n-alkane mixtures: Coarse-grained molecular dynamics simulations and diffusion-ordered spectroscopy nuclear magnetic resonance
Source: Sci Rep. 2019 Jan 30;9:1002. doi: 10.1038/s41598-018-37799-7 (PMC6353884; doi:10.1038/s41598-018-37799-7)
Supplement: Supplementary file 1 — Supplementary Material [file 41598_2018_37799_MOESM1_ESM.pdf]

Supporting Information

Fluid-solid phase transition of n-alkane mixtures: Coarse-grained molecular dynamics simulations and diffusion-ordered spectroscopy nuclear magnetic resonance

S. Shahruddin, G. Jiménez-Serratos, G.J.P. Britovsek, O.K. Matar, E.A. Müller

Molecular Model

The molecular model for n-alkanes in this study is a coarse-grained (CG) model proposed by Rahman *et al.*,[1] where n-alkanes with 3*n* (for *n* = 1, 2, …) carbons are represented as *m (=n)* spherical segments linearly and tangentially connected. The dispersion force-fields are modelled as Mie potentials (or generalised Lennard-Jones), with parameters obtained via the Statistical Associating Fluid Theory[2] (SAFT-γ) approach. A differentiation between the middle and the terminal groups (i.e. the -$CH_2CH_2CH_2$- and the $CH_2CH_2CH_3$ groups) was made to improve representability, transferability, and robustness. In Figure S1, a schematic representation of an n-alkane and the corresponding CG representation are provided.

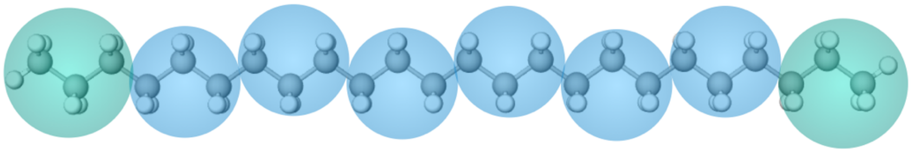

Figure S1: Cartoon of the molecular CG model for tetracosane, $C_{24}H_{50}$. Each bead represents three underlying carbon atoms and their corresponding hydrogen atoms, the fully-atomistic representation is drawn as a reference. Green 'End' beads (E) differ from the blue 'Middle' (M) beads.

The Mie potential is expressed as

$$u_{kl}^{\mathrm{Mie}}(r_{kl}) = C_{kl}\varepsilon_{kl}\left[\left(\frac{\sigma_{kl}}{r_{kl}}\right)^{\lambda_{kl}^{\mathrm{r}}} - \left(\frac{\sigma_{kl}}{r_{kl}}\right)^{\lambda_{kl}^{\mathrm{a}}}\right], \quad (1)$$

where r_{kl} is the centre-to-centre distance between segments k and l, ε_{kl} is the potential energy depth, σ_{kl} is the size scale, defined loosely as the segment diameter, and $\lambda_{kl}^{\mathrm{r}}$ and $\lambda_{kl}^{\mathrm{a}}$ are the

repulsive and attractive exponents, respectively. The latter one has been fixed in this work to a value of 6, to represent London dispersion forces. The constant C_{kl} is defined as

$$C_{kl} = \frac{\lambda_{kl}^{\mathrm{r}}}{\lambda_{kl}^{\mathrm{r}} - \lambda_{kl}^{\mathrm{a}}} \left(\frac{\lambda_{kl}^{\mathrm{r}}}{\lambda_{kl}^{\mathrm{a}}} \right)^{\lambda_{kl}^{\mathrm{a}} / (\lambda_{kl}^{\mathrm{r}} - \lambda_{kl}^{\mathrm{a}})} . \tag{2}$$

The SAFT-γ Mie parameters are fitted to vapor pressure and saturated liquid density data properties of small alkanes (C_6 to C_{12}). The unlike interactions for the end (E) and middle (M) beads are obtained from combining rules for the unlike diameters and repulsive exponent,

$$\sigma_{kl} = \frac{\sigma_{kk} + \sigma_{ll}}{2} \tag{3}$$

$$\lambda_{kl}^{\mathrm{r}} - 3 = \sqrt{(\lambda_{kk}^{\mathrm{r}} - 3)(\lambda_{ll}^{\mathrm{r}} - 3)}, \tag{4}$$

while the unlike energy parameter was directly tuned in the development of the model. In Table S1, the intermolecular force-fields obtained via the SAFT-γ equation of state, and the unlike parameters, are provided.

Table S1: Pairwise and cross-interaction parameters for n-alkanes derived from the SAFT-γ-Mie EoS[2].

Parameters	End Bead (E)	Mid Bead (M)	Mid-End bead (M-E)
σ (Å)	4.5012	4.1840	4.3426
Λ	15.947	16.433	16.188
ε/k_B (K)	358.37	377.14	345.72

The intramolecular interactions were determined using atomistic simulations as benchmarks, adopting a united atom (UA) representation, in which hydrogens are implicitly considered in the model. Monodisperse systems of n-alkanes containing $3n$ carbons (where n = 2, 3, and 4) were simulated to obtain the target bond-length and angular distributions. The intramolecular CG potentials were expressed as

$$\frac{U_{intra}}{k_{\mathrm{B}}} = \sum_{\mathrm{bonds}} \frac{1}{2} \frac{k_{\mathrm{bond}}}{k_{\mathrm{B}}} (r - r_o)^2 + \sum_{\mathrm{angles}} \frac{1}{2} k_{\mathrm{angle}} (\theta - \theta_o)^2 \quad (1)$$

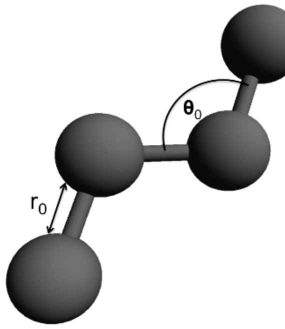

where k_{bond} and k_{angle} are the stretching and bending constants respectively, r and θ correspond to the bond length and angle, while the subscript *o* denotes the equilibrium values. The averaged values reported by Rahman *et al.*[Error! Bookmark not defined.], k_{bond} / k_{B} = 6666 K Å^{-2} with $r_o = \sigma_{kl}$, and k_{angle} / k_{B} = 2318 K rad^{-2} with θ_o= 159°, are used here.

Surrogate model mixture

Waxy crude oil originating from the Asia Pacific region under PETRONAS Operations was analysed by High-Temperature Gas Chromatography (HTGC) and SARA analysis to obtain its compositional properties. The compositional distribution analysis was performed in accordance to ASTM D7169[3] analyzing up to a carbon number of C_{120}. The chromatographic spectrum is then compared with reference standard hydrocarbons for component identification. Weight percent (wt %) of carbon distribution is calculated from the area count of each component. The HTGC analysis resulted in a carbon number distribution in the range of C_5 to C_{66}. Carbon numbers between C_{18} and C_{32} are the major components with distribution between 3.2 to 8.8 wt %. The heavy compounds were less than 1.0 wt %, while those lower than C_{18} were less than 2.0 wt %. Upon the presumption that the carbon distribution of the saturate fraction follows that of the entire crude, a discrete model consisting of eight representative components is employed. Several alkane cuts were grouped together to represent the short, middle and long chained alkanes. The composition was fixed mimicking the compositional distribution obtained from the HTGC analysis. Table S2 below includes the alkane components and their respective composition. The chemicals were purchased from Sigma Aldrich and were used without further purification. In Table S3, the composition details of the model mixture studied in the MD simulations is presented.

Table S2. Chemicals used in the model system.

Component Grouping	Component Representation (surrogate molecule)	Weight %	CAS Number	Chemical Purity (%)	Melting Point (K / °C)
C_{10-13}	Dodecane, $C_{12}H_{26}$	10.03	112-40-3	≥ 99	263.59 / - 9.5
C_{14-16}	Pentadecane, $C_{15}H_{32}$	11.24	629-62-9	≥ 99	283.15 / 10
C_{17-19}	Octadecane, $C_{18}H_{38}$	14.43	593-45-3	99	301.35 / 28.2
C_{20-22}	Heneicosane, $C_{21}H_{44}$	10.84	629-94-7	98	313.65 / 40.5
C_{23-25}	Tetracosane, $C_{24}H_{50}$	13.20	646-31-1	99	323.75 / 50.6
C_{26-28}	Heptacosane, $C_{27}H_{56}$	16.17	593-49-7	≥ 98	332.65 / 59.5
C_{29-31}	Triacontane, $C_{30}H_{62}$	14.32	638-68-6	98	339.05 / 65.9
C_{32-36}	Tritriacontane, $C_{33}H_{68}$	9.78	630-05-7	98	343.4 / 70.25

Table S3. Composition of the molecular model (mixture). *m* is the number of beads in the n-alkane CG model, which correspond to 2 end beads and (*m* -2) middle beads.

Component (surrogate molecule)	*m*	Number of molecules
Dodecane, $C_{12}H_{26}$	4	1379
Pentadecane, $C_{15}H_{32}$	5	1240
Octadecane, $C_{18}H_{38}$	6	1328
Heneicosane, $C_{21}H_{44}$	7	856
Tetracosane, $C_{24}H_{50}$	8	913
Heptacosane, $C_{27}H_{56}$	9	995
Triacontane, $C_{30}H_{62}$	10	793
Tritriacontane, $C_{33}H_{68}$	11	493

Molecular Simulation Details

Molecular dynamics simulations in this work are performed using HOOMD-blue (Highly Optimized Object-oriented Many-particle Dynamics)[4]. Randomly generated configurations at low density (to avoid molecules overlapping) are compressed at isobaric-isothermal conditions (*NPT*, constant number of particles, pressure, and temperature) at a pressure of 1 bar to generate a stable liquid phase. These simulations were run for at least 10 ns using a timestep of 0.01 ps. The following MD simulations are performed in the canonical ensemble (*NVT*, constant number of particles, volume, and temperature) exploring temperatures above the presumed melting point for the corresponding system, to calculate the

diffusion coefficients and order parameters. The *NVT* simulations are accomplished using the Martyna–Tobias–Klein (MTK) integration of the equations of motion[5,6] based on the Nosé–Hoover thermostat[7,8] while the *NPT* simulation is performed using the MTK barostat–thermostat[6,9,10]. The simulations are run for a minimum of 50 ns (longer simulation times were recorded for equilibration at lower temperatures) and a potential cut-off distance of 20 Å (~ 4.5 σ). The ensemble averages are taken from configurations generated within the final 40 ns.

Pure n-alkane system: $C_{24}H_{50}$ and $C_{30}H_{62}$

Pure-alkane monodisperse systems for tetracosane, $C_{24}H_{50}$, and triacontane, $C_{30}H_{62}$, are simulated in cubic boxes, $L_x = L_y = L_z$. Both systems contain 1639 chains, which corresponds to 13112 and 16390 CG segments in the tetracosane and in the triacontane systems, respectively. The simulations are performed by changing the temperature from 400 K to 200 K in the *NPT* and *NVT* ensembles, as described above.

8-alkane Mixture Model System

The simulation box for the 8-alkane mixture was generated randomly with the composition given in Table S2, with a total of 55288 beads (5516 beads of $C_{12}H_{26}$, 6200 beads of $C_{15}H_{32}$, 7968 beads of $C_{18}H_{38}$, 5992 beads of $C_{21}H_{42}$, 7304 beads of $C_{24}H_{50}$, 8955 beads of $C_{27}H_{56}$, 7930 beads of $C_{30}H_{60}$ and 5423 beads of $C_{33}H_{68}$) in a cubic box $L_x = L_y = L_z$. The simulations are performed by gradually reducing the temperature from 400 K to 200 K in the *NPT* and *NVT* ensembles, as described above.

Diffusion coefficients from MD simulations

The diffusion coefficient is employed as a measure of the inherent mobility of the species in solution. It is directly related to the mean square displacement (*MSD*), which is the average distance that a given particle in a system travels,

$$MSD(t) =< r^2(\Delta t) > =< |\mathbf{r}\,(t) - \mathbf{r}\,(t_0)|^2 >, \quad (6)$$

where **r**(t) is the position vector in the XYZ space at time t, and the angular brackets indicate an ensemble average, *i.e.*, an average over all particles in the system and over all possible values of the time origin, indicated as t_0. From the Einstein relation, the diffusion coefficient in a 3-dimensional system is defined as

$$D = \frac{1}{6} \lim_{t \to \infty} \frac{<[\mathbf{r}(t)-\mathbf{r}(t_0)]^2>}{t}. \tag{7}$$

In this work, the diffusion coefficient is calculated from the slope in the long-time regime of the *MSD* curve as a function of time, using the individual CG segment positions along the simulation trajectory. Following the algorithm in References[11,12], every step of the first half of data is chosen as the initial point of the calculation, $\mathbf{r}(t_0)$, and the position changes are stored for every time difference, $\Delta t=t-t_0$, from $\Delta t = 0$ to $\Delta t = t_{\text{tot}}/2$, being t_{tot} the total time of the trajectory taken for averages. In this way, the *MSD vs.* time plot spans only from 0 to $t_{\text{tot}}/2$ instead of the usual t_{tot} value, but the resulting curve presents a higher quality, since every histogram in the calculation contains the same statistics. The procedure is schematically presented in Figure S2. In contrast, when a single starting point is taken, the last histogram contains just one value: $\mathbf{r}(t_{\text{tot}}) - \mathbf{r}(0)$. This is also presented schematically in Figure S2, for comparison. The improvement in the quality of the *MSD* long-time behaviour enables a better fitting of the slope, which is applied to the last 20 or 25 % of the points in the curve, avoiding the short-time diffusion regime. In the 50 ns simulations, the positions of the segments are stored every 0.1 ns during the last 40 ns, so that the total time of the simulation for the averages is $t_{\text{tot}} = 40$ ns, and the position differences are calculated for t_0 values from 0.1 to 20 ns.

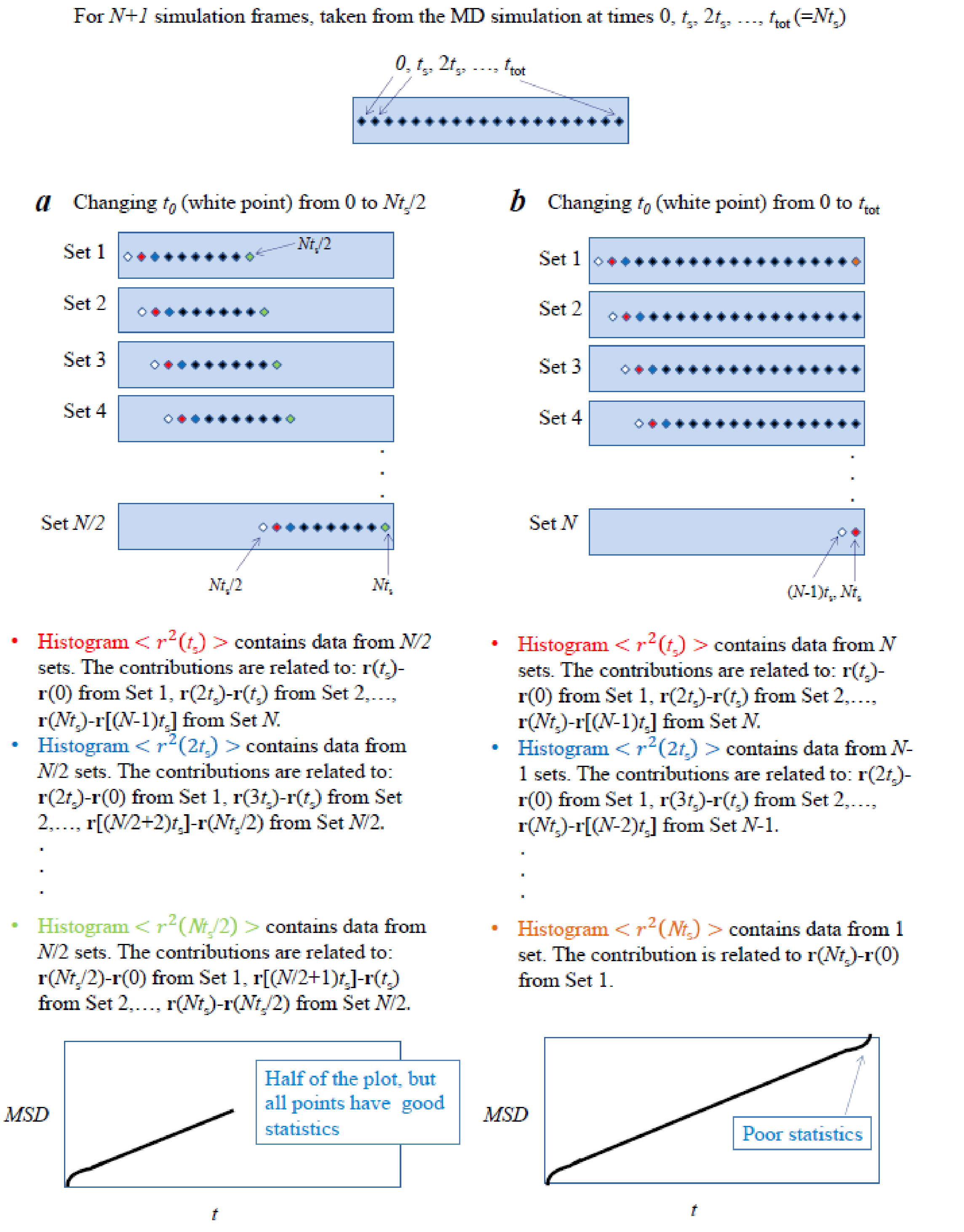

Figure S2: Schematic representation of two algorithms tested to obtain the *MSD vs* time curves. The time origin t_0 is changed to improve statistics in the analysis, following algorithm (*a*) or (*b*). The data is arranged into sets for every possible value of t_0. The algorithm labelled as (*a*) was chosen to obtain the diffusion coefficients in this work. A schematic example of the outputs for each case is also given.

End-to-end distances and radius of gyration from MD simulations

Order parameter analysis is also applied to complement the study of the changes that occur with the alkanes during the transition process in the MD simulations. The end-to-end distance and radius of gyration of the n-alkanes in neat and mixture form were analysed to evaluate the molecule's flexibility. In Figure S3, the end-to-end distance and radius of gyration for $C_{12}H_{26}$, $C_{24}H_{50}$, and $C_{30}H_{62}$ in the 8-alkane mixture are presented as a function of temperature. Both properties increase with the carbon number, as expected, and both properties increase as the temperature is decreased. The general trends are consistent with the ones reported in the literature[1314]. The chains appear to be more stretched or extended at a lower temperature, which would improve the alignment between chains in the system towards the glassy state. It is interesting to note that $C_{12}H_{26}$, being the smallest alkane in the system, has a minimum variation in the structural properties as a function of the temperature, while the change in longer chains, $C_{24}H_{50}$ and $C_{30}H_{62}$, is more evident. Further comparison of the simulation results is made for the end-to-end distance and the radius of gyration of n-alkanes in their neat form and in a mixture, as shown in Figure S4. It is found that there is no significant difference in both systems, suggesting that the structural changes of the n-alkanes are not governed by the mixture.

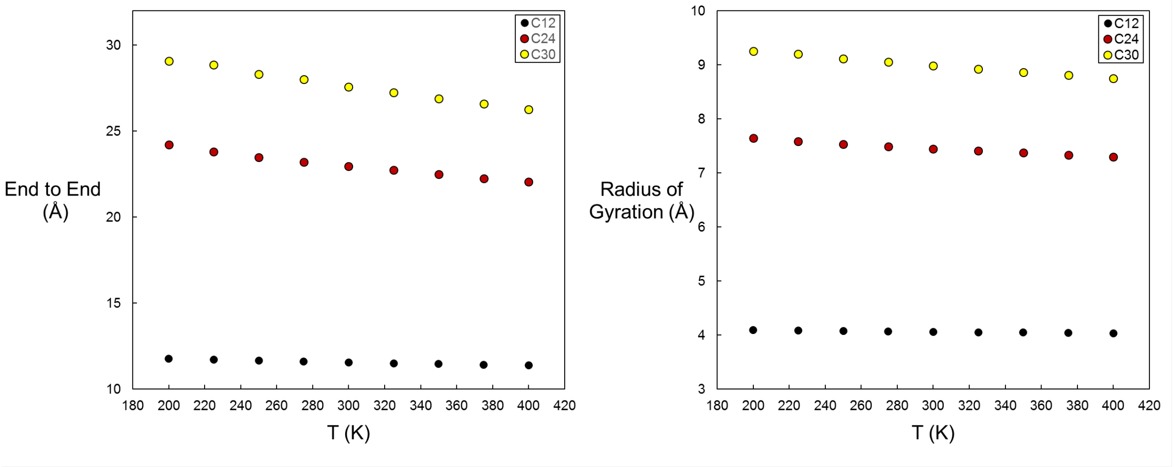

Figure S3: End-to-end distance and radius of gyration for $C_{12}H_{26}$, $C_{24}H_{50}$ and $C_{30}H_{62}$ (denoted by the solid black, red and yellow circle markers) in the 8-alkane mixture determined from NVT simulations.

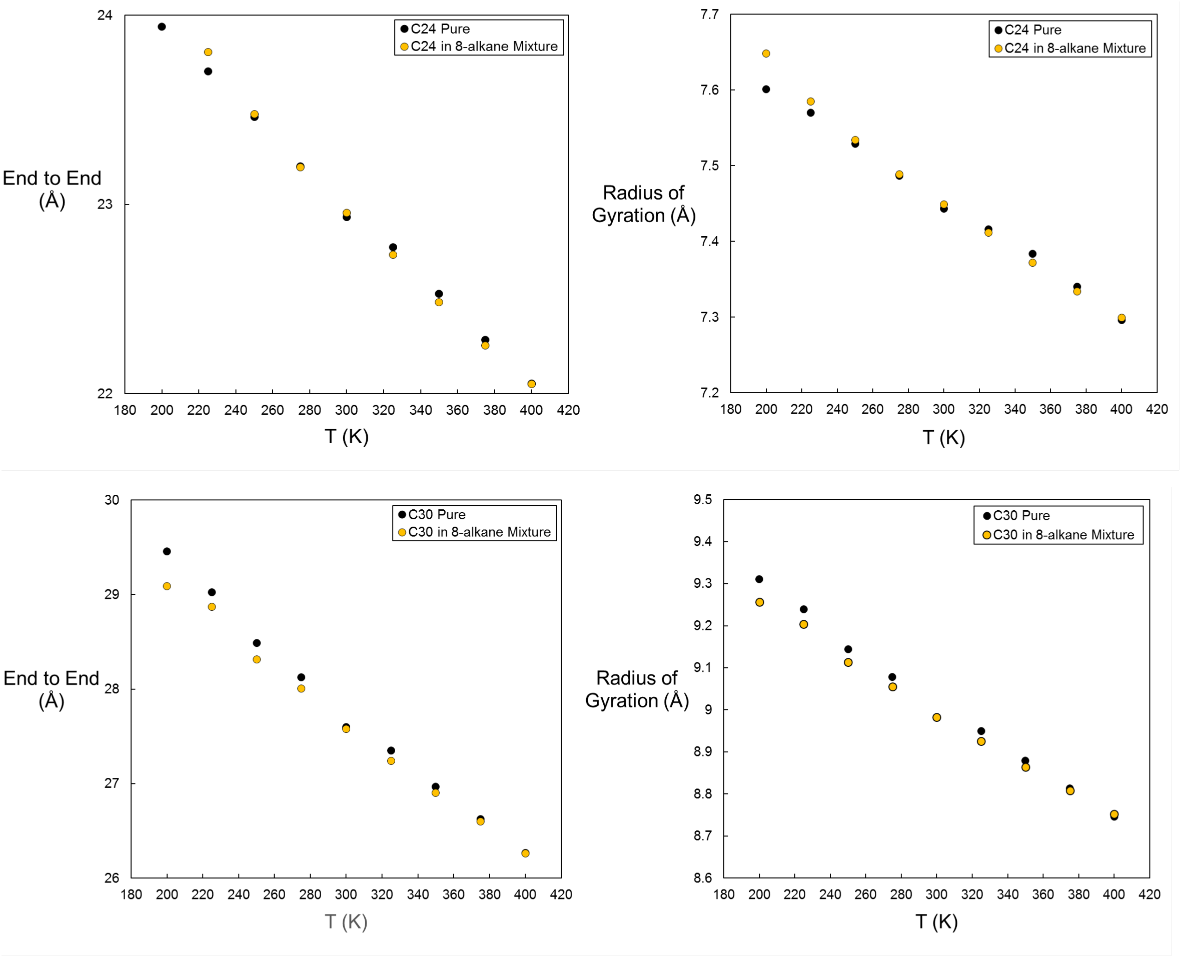

Figure S4: Comparison of end-to-end distance and radius of gyration for $C_{24}H_{50}$ and $C_{30}H_{62}$ in their neat form and in the 8-alkane mixture determined from MD simulations at *NVT* conditions.

Diffusion-Ordered Spectroscopy (DOSY) Nuclear Magnetic Resonance (NMR)

DOSY experiments were performed on a Bruker 500MHz AVANCE III HD spectrometer running TopSpin3.2 and equipped with a z-gradient bbfo/5mm tuneable SmartProbe and a GRASP II gradient spectroscopy accessory providing a maximum gradient output of 53.5G/cm (5.35G/cmA). The 1H DOSY spectra were collected using the Bruker pulse program ledbpgp2s at a frequency of 500.13MHz with a spectral width of 5500Hz (centred on 4.5ppm) and 32768 data points. A relaxation delay of 12s was employed along with a diffusion time (large delta) of 150ms and a longitudinal eddy current delay (LED) of 5ms. Bipolar gradients pulses (little delta/2) of 5ms and homospoil gradient pulses of 1.1ms were used. The gradient strengths of the 2 homospoil pulses were -17.13% and -13.17%. 24 experiments were collected with the bipolar gradient strength, initially at 2% (1st experiment), linearly increased to 95% (24th experiment). All gradient pulses were smoothed-square shaped (SMSQ10.100)

and after each application a recovery delay of 200us used. The data was processed using 16384 data points in the direct dimension applying an exponential function with a line broadening of 1Hz and 128 data points in the indirect dimension. Further processing was achieved using the Bruker Dynamics Center software (version 2.1.7) – error estimation by Monte Carlo simulation with a confidence level of 95%[15,16,17].

Differential Scanning Calorimetry (DSC)

DSC is used in this work to study the thermal transition of the polymer. All measurements were carried out on a TA Instrument DSC Q20. A small amount of sample (approximately 2-5mg) was weighed in a Tzero aluminium pan and clamped with a sample lid. Three heating/cooling cycles were performed of which data from the second cycle was used for analysis with the TA Universal Analysis Software. A heating rate of 10 °C/min was applied for -100 to 200 °C temperature range. The melting temperature was extracted from the endothermic peak and the glass transition temperature T_g was determined based on the inflexion point of the change in the heat capacity.

This phase transition observed by DOSY-NMR is also verified with conventional DSC analysis where the melting point of $C_{24}H_{50}$ and $C_{30}H_{62}$ were determined using the heat-cool-heat cycle methodology to correctly measure the phase transition. Figure S5 shows the melting point for $C_{24}H_{50}$ and $C_{30}H_{62}$ determined by DSC, which is consistent with the melting behavior reported elsewhere[18]. Two peaks were recorded for both alkanes, and this observation is also consistent with other works in the literature, where higher alkanes were generally reported to have two peaks recorded. The true melting point is recorded at the main peak, which is usually at a higher temperature than the second peak. The appearance of the second peak corresponds to a solid-solid transition between a rotator phase and the more common crystal[19]. The melting curve for the 8-component alkane mixture is shown in Figure S6, where there is evidence of a spread in the signal and a very ambiguous result if one is expected to report a unique temperature for the transition. While the standard analysis reports a melting point of 38.6 °C, it is evident from the thermogram that the melting point (WAT) becomes ill-defined and would spread from 32°C to 40°C, approximately.

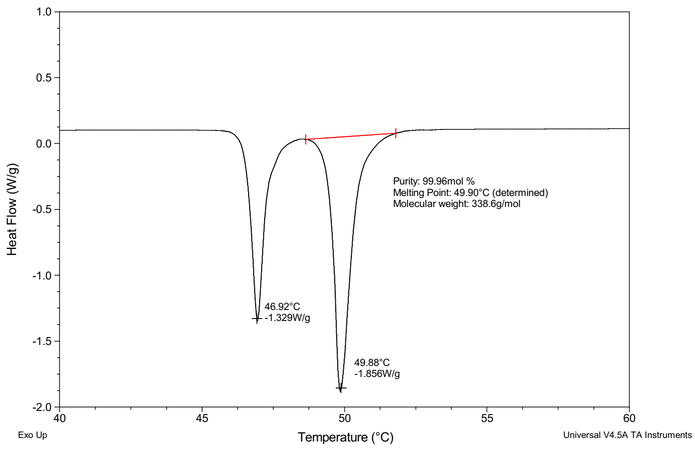

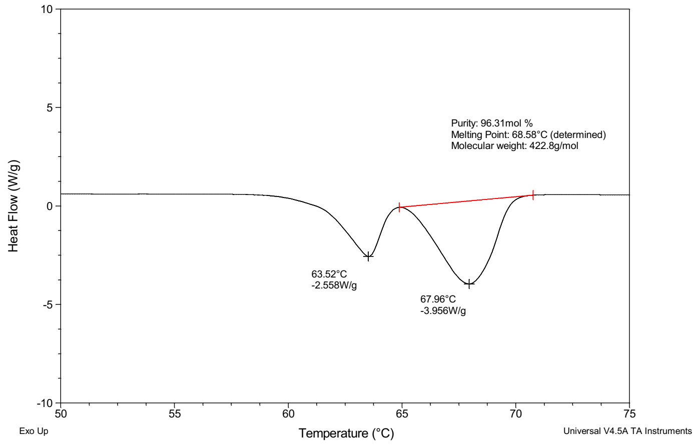

Figure S5: Melting points of $C_{24}H_{50}$ (top) and $C_{30}H_{62}$ (bottom) determined by DSC using the heat-cool-heat cycle methodology in a hermetic pan under Helium, at 10 °C/min.

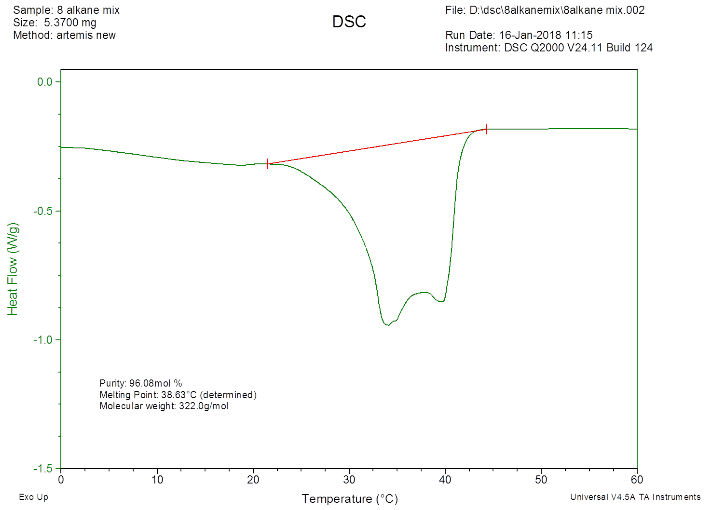

Figure S6: Melting point for the 8-alkane mixture (38.6 °C) determined by DSC using the heat-cool-heat cycle methodology in a hermetic pan under Helium, at 10 °C/min.

Diffusion results for C-30

Figure S7 shows the results of the diffusion coefficient for n-C-30 (CG and DOSY) as compared to the results of united atom models and other NMR data. Comments are similar to those surrounding Figure 2 of the main text.

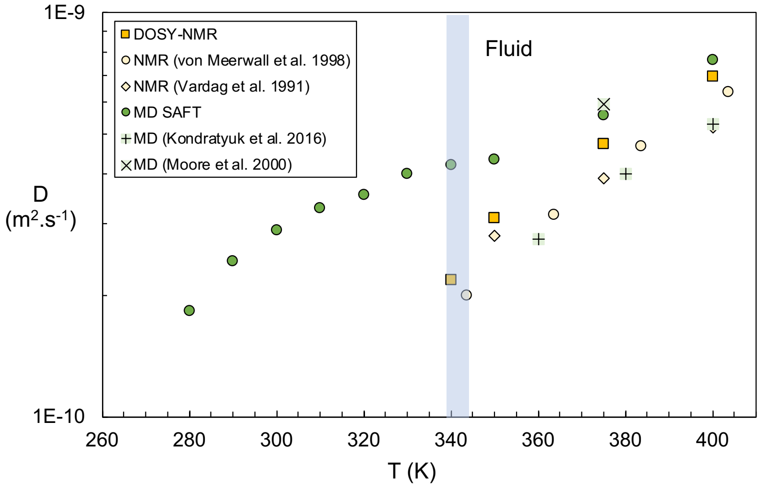

Figure S7: Diffusion coefficients of n- $C_{30}H_{62}$ as a function of temperature as obtained from DOSY NMR (yellow squares) and coarse-grained MD simulations using the SAFT force field (green circles). The comparison is made with MD data using UA models (green crosses[43], exes[42] and PFGSE NMR (yellow circles [44], diamonds[20] [ref]}

Melting point for C-24

For the results shown in figure 1 (melting of a solid), we chose to construct the crystal by aligning stretched-out molecules packed in a primitive orthorhombic structure. The resulting solid is equilibrated at 200 K and a 1 bar in the NσT ensemble, allowing the different dimensions of the solid to accommodate to the closest packing. The resulting structure is temporally stable, displays translational order and has a higher density than its liquid counterpart.

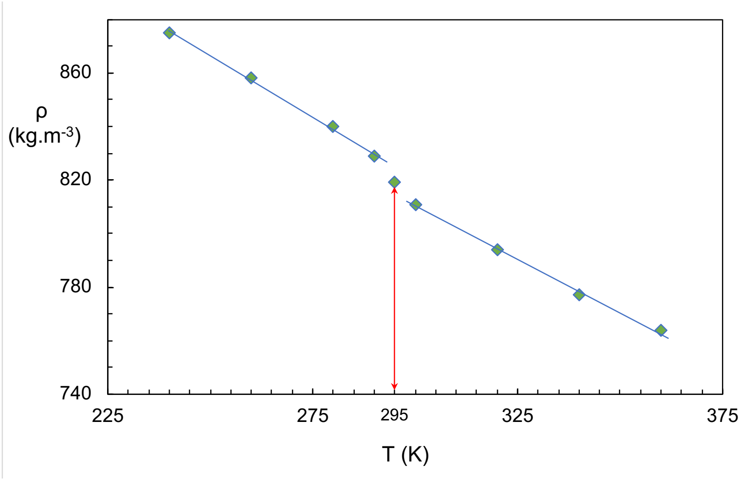

Figure S8: Detail of Figure 1, showing the density of pure n-$C_{24}H_{50}$ as a function of temperature. Green diamonds correspond to results from simulations of melting of a pre-assembled crystal. Blue lines are trendlines of the densities of the solid phases (left) and liquid phases (right). Red arrow marks the assigned melting point.

Experimental diffusion data

Raw NMR data is reported in table S4. All measurements are repeated three times. Error bars are calculated based on the NMR average values.

Table S4. Raw experimental data.

Temperature (K)	$C_{24}H_{50}$		$C_{30}H_{62}$		8 Alkane Mixture	
	Diffusion (m^2/s)	Error (%)	Diffusion (m^2/s)	Error (%)	Diffusion (m^2/s)	Error (%)
400	1.31E-09*	6.51	6.96E-10	4.02	1.45E-09	4.14
375	6.69E-10	1.79	4.72E-10	0.21	1.08E-09	4.63
350	4.78E-10	1.05	3.11E-10	0.48	6.81E-10	3.01
340			2.18E-10**	1.15		
325	1.99E-10	0.25			4.00E-10	5.38

* Outlier. Suspect data point.

** Data point very close to melting point.

References

[1] Rahman, S., Lobanova, O., Jiménez, M. G., Braga, C., Raptis, V., Müller, E. A., Jackson, G., Avendaño, C., and Galindo, A. SAFT-γ Force Field for the simulation of molecular

fluids. 5. Hetero-group coarse-grained models of linear alkanes and the importance of intramolecular interactions. *J. Chem. Phys.* (2018). DOI: 10.1021/acs.jpcb.8b04095
[2] Müller, E. A., and Jackson, G. Force-Field Parameters from the SAFT-γ Equation of State for Use in Coarse-Grained Molecular Simulations. *Annu. Rev. Chem. Biomol. Eng.* **5**, 405–427 (2014).
[3] ASTM D7169-11: Standard Test Method for Boiling Point Distribution of Samples with Residues Such as Crude Oils and Atmospheric and Vacuum Residues by High Temperature Gas Chromatography. *ASTM Int.* 1–17 (2011). doi:10.1520/D7169.
[4] Anderson, J. A., Lorenz, C. D., and Travesset, A. General purpose molecular dynamics simulations fully implemented on graphics processing units. *J. Comput. Phys.* **227**, 5342–5359 (2008).
[5] Cao, J., and Martyna, G. J. Adiabatic path integral molecular dynamics methods. II. Algorithms. *J. Chem. Phys.* **104**, 2028–2035 (1996).
[6] Martyna, G. J., Tobias, D. J., and Klein, M. L. Constant pressure molecular dynamics algorithms. *J. Chem. Phys.* **101**, 4177–4189 (1994).
[7] Nosé, S. A unified formulation of the constant temperature molecular dynamics methods. *J. Chem. Phys.* **81**, 511 (1984).
[8] Hoover, W. G. Canonical dynamics: Equilibrium phase-space distributions. *Phys. Rev. A* **31**, 1695–1697 (1985).
[9] Yu, T. Q., Alejandre, J., López-Rendón, R., Martyna, G. J., and Tuckerman, M. E. Measure-preserving integrators for molecular dynamics in the isothermal-isobaric ensemble derived from the Liouville operator. *Chem. Phys.* **370**, 294–305 (2010).
[10] Tuckerman, M. E., Alejandre, J., López-Rendón, R., Jochim, A. L., and Martyna, G. J. A Liouville-operator derived measure-preserving integrator for molecular dynamics simulations in the isothermal-isobaric ensemble. *J. Phys. A. Math. Gen.* **39**, 5629–5651 (2006).
[11] Haile, J.M. *Molecular Dynamics Simulation*. John Wiley & Sons, Inc., New York (1992).
[12] Keffer D. The working man's guide to obtaining self-diffusion coefficients from molecular dynamics simulations (2001). Available from: http://www.cs.unc.edu/Research/nbody/pubs/external/Keffer/selfD.pdf.
[13] Alatas, P. V., Tsalikis, D. G., Mavrantzas, V. G. Detailed molecular dynamics simulation of the structure and self-diffusion of linear and cyclic n-alkanes in melts and blends. *Macromol. Theory Simul.* **26**, 201600049 (2016).
[14] Dimitriou, C. J., McKinley, G. H., Venkatesan, R. Rheo-PIV analysis of the yiending and flow of model waxy crude oils. *Energy and Fuels* **25 (7)**, 3040–3052 (2011).
[15] Kerssebaum, R., and Salnikov, G. DOSY and Diffusion by NMR. *Topspin - Bruker* (2006).
[16] Johnson, C. S. Diffusion ordered nuclear magnetic resonance spectroscopy: principles and applications. *Prog. Nucl. Magn. Reson. Spectrosc.* **34,** 203–256 (1999).
[17] Durand, E., Clemancey, M., Lancelin, J.-M., Verstraete, J., Espinat, D., and Quoineaud, A.-A. Effect of chemical composition on asphaltenes aggregation. *Energy and Fuels* **24,** 1051–1062 (2010).
[18] Hammami, A., and Mehrotra. A.K. Thermal behavior of polymorphic n-alkanes: effect of the cooling rate on the major transition temperatures. *Fuel*, **74**, 96-101 (1995).
[19] Corsetti, S., Rabl, T., McGloin, D., Kiefer, J. Intermediate phases during solid to liquid transitions in long-chain n-alkanes. *Phys. Chem. Chem. Phys.*, **19**, 13941–13950 (2017).
[20] Vardag, T., Karger, N. & Ludemann, H. D. Temperature and Pressure Dependence of Self Diffusion in Long Liquid n-Alkanes. *Ber. Bunsen. Phys. Chem.* **95**, 859–865 (1991).
